# Comparison between sand and clay clogging mechanisms of pervious concrete pavement

**DOI:** 10.1038/s41598-022-13483-9

**Published:** 2022-06-03

**Authors:** Yunkang Rao, Haiying Fu, Tao Yang, Huailin Chen, Zhe Zhang, Haojiang Ding

**Affiliations:** 1 Department of Geotechnical Engineering, School of Civil Engineering, Southwest Jiaotong University, No.111, North Section 1, Erhuan Road, Chengdu, 610031 Sichuan China; 2 China Railway Eryuan Engineering Group Co. Ltd, Chengdu, 610031 China

**Keywords:** Civil engineering, Environmental impact

## Abstract

Pervious concrete (PC) pavement has been widely accepted as a green infrastructure but is prone to clogging. This study comparatively investigated sand and clay clogging mechanisms of PC and vertical sediment distributions of sand-clogged and clay-clogged PCs. Clay and three sizes of sand were used to clog PC under two exposure methods (no drying and drying). X-ray computed tomography (CT) was used to scan the clogged samples before and after 30 pressure washing cycles. The clogged permeability and permeability after each washing cycle were measured. The clogging patterns of sand depend mainly on sand particle sizes relative to PC pore sizes. The applied fine sand, coarse sand, and medium sand cause easy-passing clogging, surface clogging, and full-depth clogging, respectively. After clay clogging, more than 77% of the total retained clay occurs within the vertical region 24–72 mm below the sample surface; the most clogging (the lowest-permeability layer) occurs at a depth of approximately 48 mm. The dried clay retained within the region 40–120 mm below the surface (especially within the lowest-permeability layer) is hard to wash away because the drying process increases the cohesion of internal clay particles and clay adhesion to the rough, tortuous pore wall of PC. The clogged normalized permeability of 0.154 and permeability recovery ratio of 4.91% in dried clay-clogged samples are lowest among all the samples. However, pressure washing readily washes away the retained undried clay. Accordingly, it is recommended that pressure washing is used to eliminate the clogging effect of dried clay before hot, sunny exposure conditions dry the retained clay. This study provides evidence for developing effective pavement maintenance strategies.

## Introduction

Pervious concrete is a novel and sustainable pavement material, which generally consists of coarse aggregates, cement, and water^1,2^. Pervious concrete (PC) pavement has many functional and environmental benefits in urban settings because it facilitates stormwater infiltration, reduces surface runoff, mitigates the heat island effect, etc^3–6^. However, the performance of PC pavement declines over time because the pores are clogged by sediment particles^7–11^.

Many studies have employed sand with different particle sizes and have explored the influence of sand clogging on the permeability of PC^12–15^. Deo, et al.^12^ employed sand with two size ranges (0.10–0.84 and 0.84–1.80 mm) as clogging materials. Cui, et al.^13^ employed three sizes of sand as clogging materials. Most recently, Nan, et al.^15^ selected sand particles smaller than 0.3, 0.3–0.6, 0.6–0.9, and 0.9–2.2 mm for PC clogging. It has been reported that sand clogging significantly reduces the permeability of PC^16,17^.

The clogging behaviour of sand without a cohesive nature is mainly affected by sand particle sizes relative to PC pore sizes^7,18^. Particles much larger than the pores are retained in the near-surface region. Finer particles tend to be trapped throughout PC. Very fine particles easily pass through PC. In contrast, clogging behaviours of clay, whose particle sizes are much smaller than those of sand and PC pores, depend more on its cohesiveness and climatic exposure conditions^19^. Generally, clay shows much lower permeability than sand, with typical infiltration rates of 210 mm/h for sand and 1 mm/h for clay^20^. Therefore, clay retained within the pores of PC may be much more damaging than sand. But it has been reported that clay is easily carried through the pores of PC because it is much smaller than the pores^17,21,22^. However, a very significant factor—drying, which frequently occurs under field conditions, is not considered by these studies.

Under field conditions, hot, sunny weather typically occurs and dries clogged pavement after rain events during which runoff water carrying sediment flows into the pavement. Accordingly, Haselbach^19^ considered drying in the clogging tests of clay, and a 50 °C oven was used to dry PC samples after performing each clogging cycle. She observed that the infiltration rate of PC clogged with bentonite clay is 70 mm/h, being only 1.15% of a 6100-mm/h unclogged value, even after four rinse cycles. This finding is significant because it confirms that the clogging of dried clay may dramatically reduce the permeability of PC and may be difficult to remove with rinse cycles. However, this study did not quantitatively measure the vertical sediment distribution or explore the clogging mechanism of dried clay in depth.

The distribution of vertical sediment is significant because it greatly affects effectiveness of cleaning methods in rehabilitating clay-clogged PC. In the field, the PC pavement needs periodic cleaning to maintain its performance. Pressure washing is one of the most recommended techniques for rejuvenating clogged pervious pavement^23,24^. Considering that pressurized water during the washing cycles is able to enter the internal structure of PC, the scouring force of washing water during pressure washing may affect clay retained within any depth of PC. However, the scouring force and cleaning effect of washing water may differ at different depths of PC^25^. Specifically, pressure washing with a high scouring force may easily wash away the particles near the surface. However, the scouring force of pressurized water flowing through regions far from the surface is lower because the skeleton of PC blocks the flow of water near the surface, so the cleaning effectiveness may be lower in regions far from the surface.

Clay particles reportedly tend to be carried through the pores of PC with water when not considering drying^17,22^. However, many studies have reported that drying increases soil cohesion and bond formation^26,27^; thus, clay adhesion to rough, tortuous pore wall of PC probably increases after drying. Accordingly, it is necessary to investigate the dried clay-clogging mechanism, which will improve our understanding of clay clogging in PC. We previously investigated the influence of drying on clay clogging, but did not compare clay clogging with sand clogging or investigate the vertical sediment distributions of sand-clogged PCs^25^.

This study aims to provide evidence for developing effective pavement maintenance strategies by comparatively investigating sand and clay clogging mechanisms of PC and vertical sediment distributions of sand-clogged and clay-clogged PCs.

## Materials and methods

Clay and three sizes of sand were used to clog PC under two exposure methods (no drying and drying). X-ray computed tomography (CT) was used to scan the clogged samples before and after 30 pressure washing cycles. The clogged permeability and permeability after each washing cycle were measured.

### Mixture proportion and sample preparation

The mixture proportion of the PC investigated herein was employed in a real pervious pavement engineering. The mixture comprised 113 kg of water, 402 kg of ordinary Portland cement, 1539 kg of aggregates, and 14 kg of admixture per cubic meter. The admixture contained a viscosity-modifying admixture, polycarboxylate-based superplasticiser, and air-entraining admixture. Only aggregates of 4.75–9.5 mm were employed.

The mixture was first uniformly mixed with a laboratory mixer and then injected into cylindrical PVC moulds with a height of 132 mm and an external diameter of 110 mm. Next, compaction was performed until a 120-mm height was reached. A constant-humidity chamber (95% relative humidity, 20 °C ± 2%) was employed to cure the samples within the PVC moulds for 28 days prior to subsequent experiments.

### Sand and clay used for clogging

The clogging experiments employed sediment soil collected from the hills adjacent to the pervious pavement. The collected soil was first dried at 105 °C for 24 h after the soil impurities were removed. Next, the soil was screened with sieves of 0.075, 0.25, 0.5, and 1 mm; thus, the soil was classified into particles of five size ranges (< 0.075, 0.075–0.25, 0.25–0.5, 0.5–1, and > 1 mm). Considering that particles that are too large cannot enter the internal pores of PC, only particles smaller than 0.075, 0.075–0.25, 0.25–0.5, and 0.5–1 mm were used in the clogging experiments to explore the influence of particle size on the PC clogging.

Before the clogging experiments, the properties of these four soils were determined according to the local standard ‘Specification of soil test’ (SL237-1999). The latter three soils were classified as sand according to ASTM D2487-11 because the particles ranged from 0.075 to 4.75 mm. In this study, these three sizes of sand (0.075–0.25, 0.25–0.5, and 0.5–1 mm) were referred to as fine, medium, and coarse sand, respectively. For the soil smaller than 0.075 mm, a clay fraction of 40.81% and silt fraction of 59.19% were determined; the moisture contents corresponding to the liquid and plastic limits were 38.09% and 20.45%, respectively. According to ASTM D2487-11, this soil was categorized as lean clay.

A critical parameter is the quantity of sand (or clay) used to clog the sample, i.e., the loading. To simulate a catastrophic event including storm surge and upslope soil fence failure, only one clogging cycle was used; hence, the large loading was required to produce measurable effects and to reveal the clogging potential of sand (or clay). However, most particles did not enter the internal pores of the samples at a very large loading. To determine the appropriate loading, preliminary trials were performed by using different quantities of sand or clay. According to preliminary trials, a suspension containing 240-g sand and 3-L water or 240-g clay and 1-L water was ultimately selected.

### Permeability test

**Figure 1:**
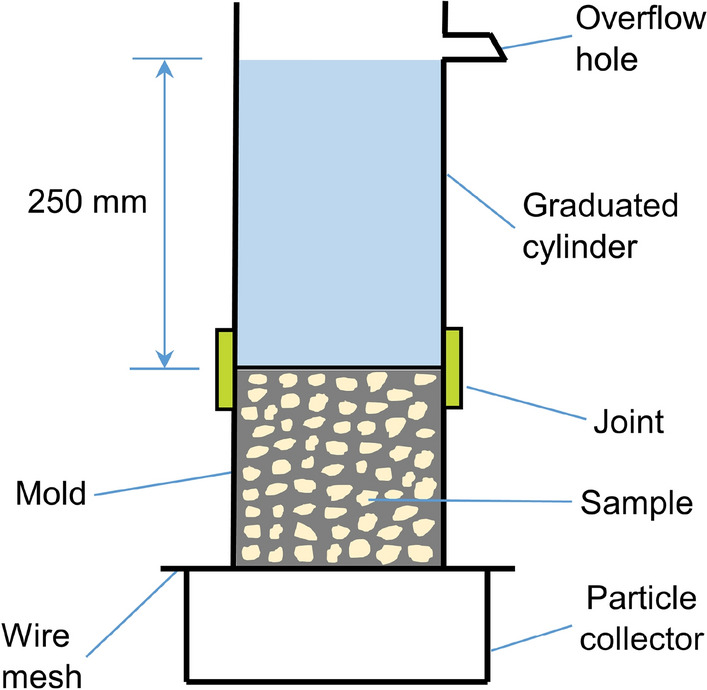
Falling head apparatus for permeability test.

A falling head method is convenient for collecting the particles traveling through the sample, so this method was used to measure the permeability of PC, as reported in previous studies^28,29^. Figure 1 shows the test apparatus. To facilitate installation and disassembly, a joint was used to connect a graduated cylinder to the sample within its mould. The connection between the cylinder and joint was sealed by using glass glue. The outside wall of the mould and the inside wall of the joint were coated with processed plasticene so that water leakage along the edge was prevented.

As shown in Fig. 1, an overflow hole was located at a height of 250 mm above the sample surface, implying a 250-mm water head difference. The permeability (*K*) was calculated according to the following equation, which was also used in previous studies^2,25^.

1 $${\text{K =}} \frac{\text{AL}}{{\text{at}}}{\text{ln}}\frac{{\text{h}}_{1}}{{\text{h}}_{2}}$$

where *a* and *A* denote the area of the sample and graduated cylinder (mm^2^), respectively; *t* denotes the time for water to pass from level ${\text{h}}_{1}$ to ${\text{h}}_{2}$ in the cylinder (s); and *L* denotes the length of the sample (mm).

### Vertical distribution of sediment

The vertical distribution of sediment in the clogged samples was determined by using CT scan, a nondestructive examination technique. Phases with different densities exhibit different X-ray absorption coefficients, which causes these different phases to show disparities in grey values in the CT images; thus, different phases can be easily distinguished by greyscale division^30,31^.

High-resolution X-ray CT was used to scan the samples. Through CT scan, numerous 2D images at different angles were obtained as the sample fixed on a rotational stage was rotated 360° while a radiation beam passed through the sample. Next, a complete 3D tomographic image was reconstructed based on these 2D images using a back-projection algorithm.

**Figure 2:**
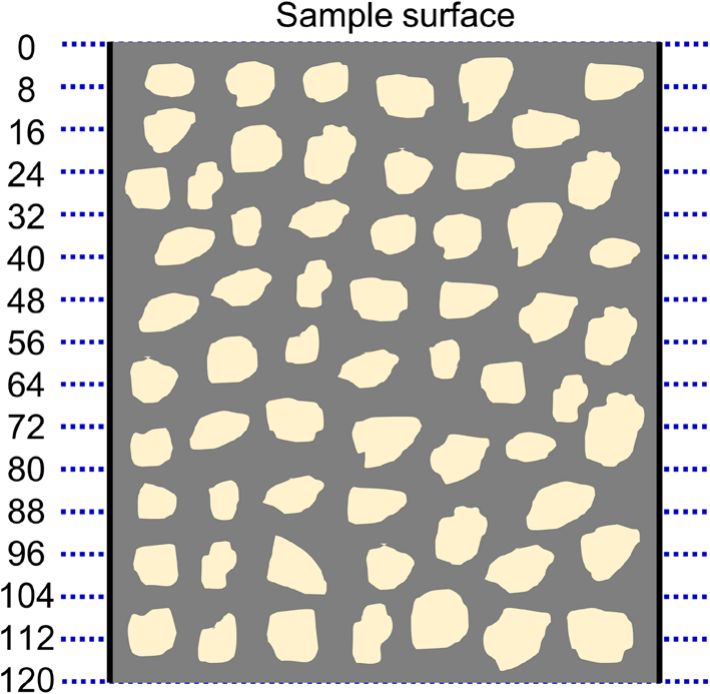
Vertical depths of 16 planar images.

To avoid disturbances from demoulding, the samples were scanned within their respective moulds. For each sample, to characterise the vertical distribution of sediment, 16 planar images at 8-mm vertical intervals (0, 8, 16, 24…120 mm from the sample surface) were chosen, as shown in Fig. 2.

**Figure 3:**
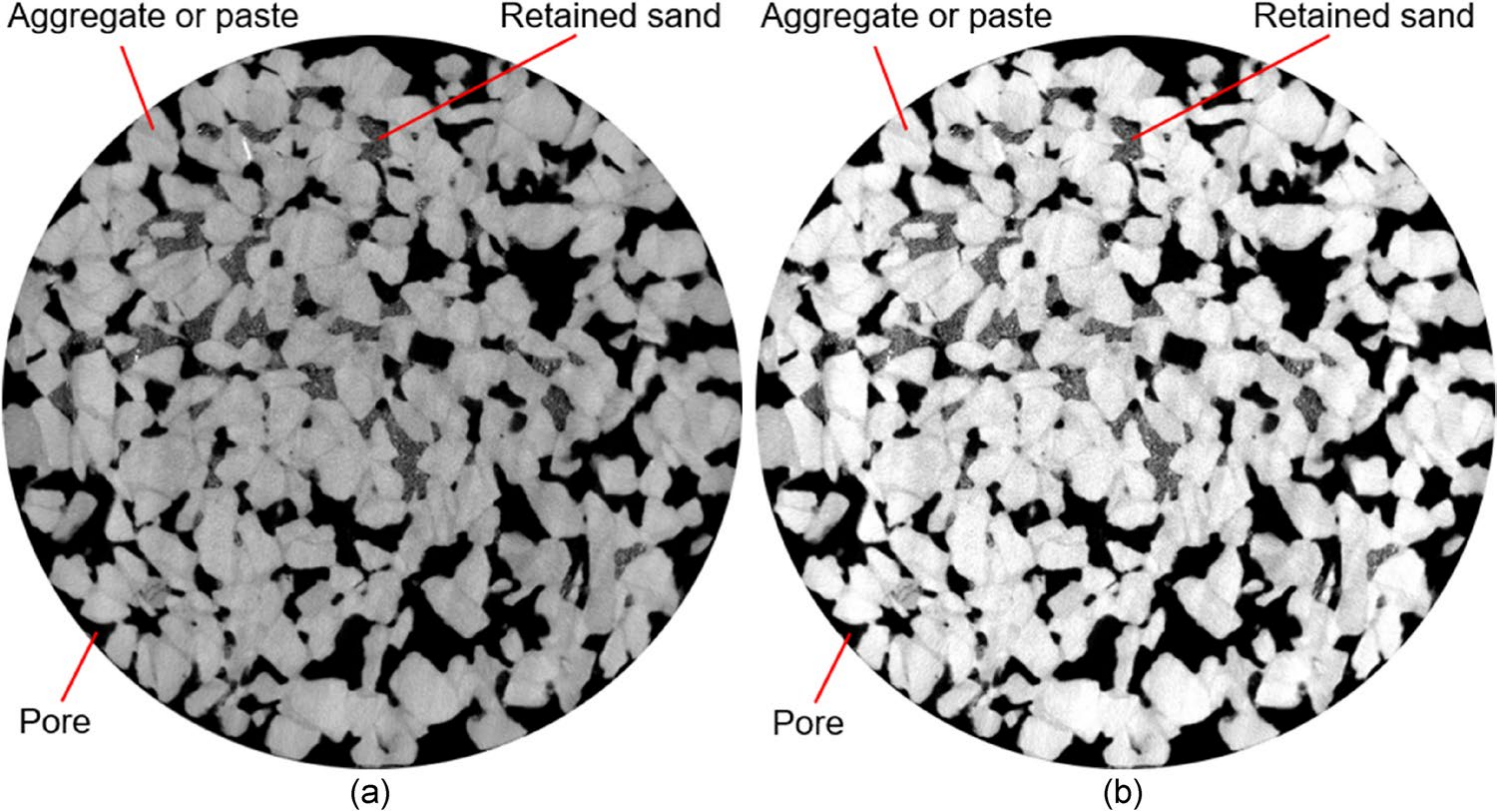
Images processing procedures for sand-clogged samples: (**a**) images cropping and (**b**) images contrast enhancement. The pore was darkest in colour, followed by the retained sand and then the aggregate (or cementitious paste).

**Figure 4:**
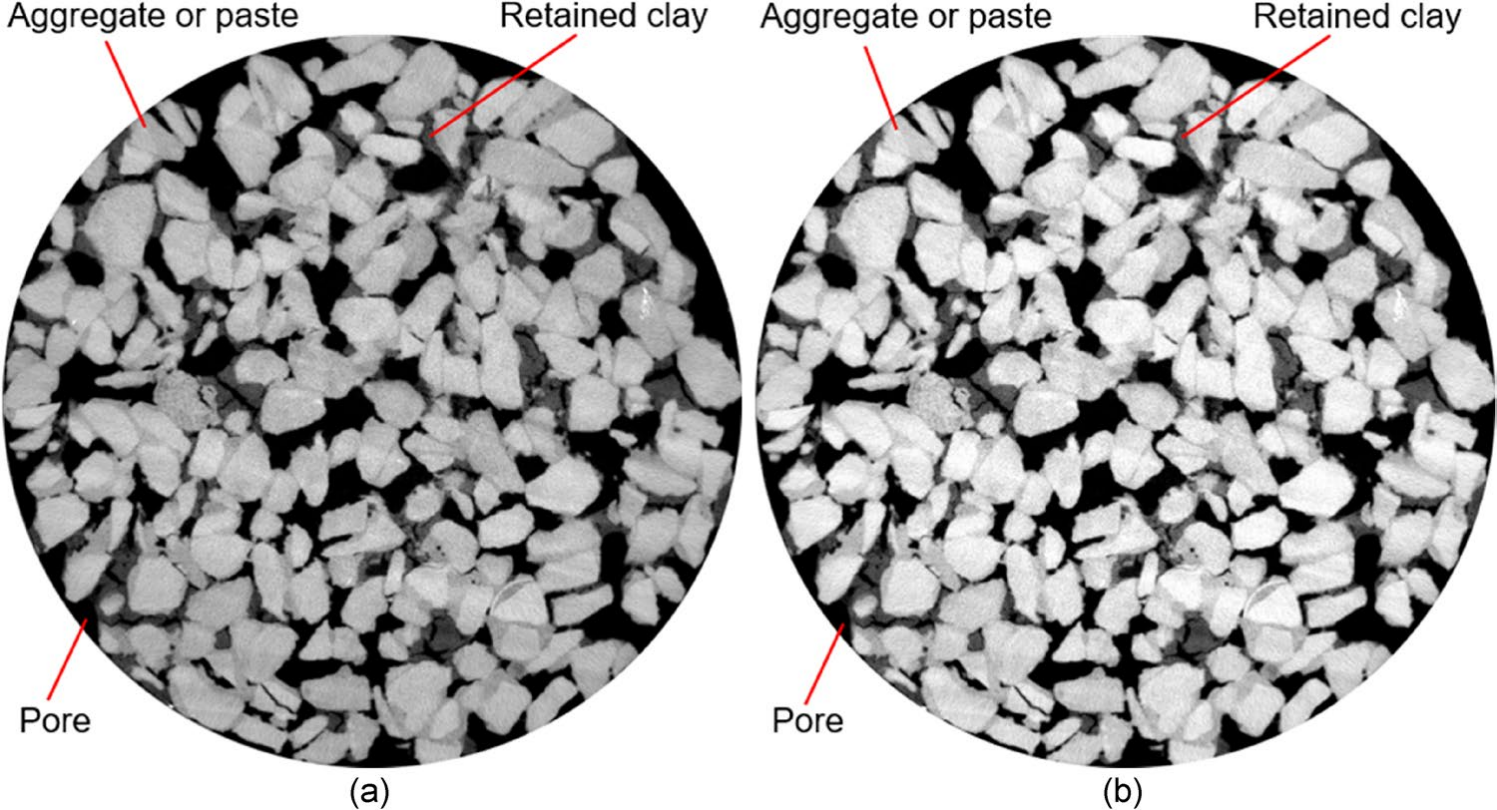
Images processing procedures for clay-clogged samples: (**a**) images cropping and (**b**) images contrast enhancement. The pore was darkest in colour, followed by the retained clay and then the aggregate (or cementitious paste).

To determine the vertical distribution of sediment, these 16 greyscale images of each sample were further processed and analysed by using image analysis software. Overall, the image processing included three procedures: image cropping, image contrast enhancement, and object selection. Each image was first cropped to remove the PVC mould, as shown in Fig. 3a or Fig. 4a. Next, the brightness contrast was adjusted to improve the recognisability of images (Fig. 3b or Fig. 4b).

The pore was darkest in colour, followed by the retained sand (or retained clay) and then the aggregate (or cementitious paste), as shown in Fig. 3 or Fig. 4. This occurs because different phases exhibit varying grey values. When the grey values of only the retained sand (or retained clay) were selected, the sand (or clay) zone was identified, and its area was then summed. By dividing the retained sand (or retained clay) area by the total area, the area fraction of the sand (or the clay) was finally calculated. As a result, the sand (or clay) area fractions of 16 greyscale images along the vertical direction were determined to profile the vertical distribution of sediment.

### Experimental procedure

**Figure 5:**
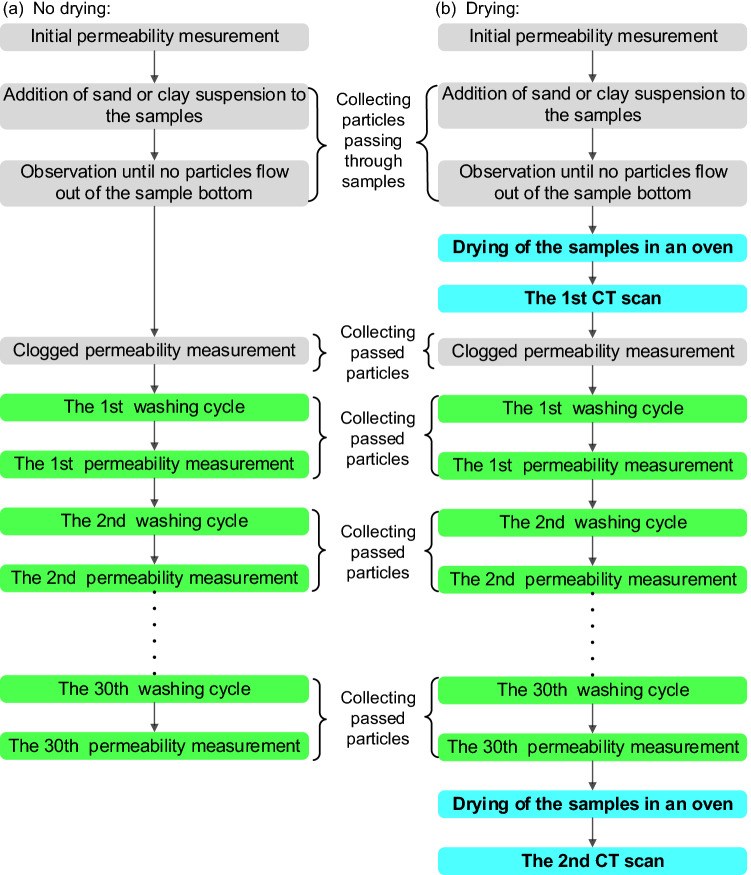
Flow diagrams of the experiments: (**a**) no drying and (**b**) drying.

To investigate the influence of drying on sand or clay clogging in PC, two exposure approaches, that is, no drying and drying, were used following the addition of sand suspension or clay suspension to clog the samples. Figure 5 shows the flow diagrams of experiments using two exposure methods. For both methods, to mimic the transport of runoff carrying soil particles, the suspension was uniformly spread on the surface to clog the samples. In the no drying-clogging experiments (Fig. 5a), 30 cycles of pressure washing were applied to undried samples after no particles flow out of the bottom of samples. In contrast, in the drying-clogging experiments (Fig. 5b), after no particles flowing out of the bottom of samples, samples were immediately dried for 24 h with a 50 °C oven, followed by 30 pressure washing cycles. The same washing time was used in each pressure washing cycle. The jet was moved regularly over the sample surface to ensure that the jet was applied as equally as possible over the entire surface area; the angle of the jet was applied at approximately 90◦ to the sample surface, with the distance between the jet and the surface being always controlled at approximately 10 cm.

Drying was the main difference between the no drying-clogging and drying-clogging experiments. The drying was intended to mimic hot and sunny field conditions, which typically occur and dry the clogged pavement after rain events. In field conditions, the drying temperature of 50 °C, which was also used in published studies^19,25^, likely occurs frequently, particularly during summer.

To determine the permeability reduction caused by clogging, the initial unclogged permeability and clogged permeability without any washing were measured, as explained in Fig. 5. Additionally, to obtain the permeability recovery caused by pressure washing, the permeability after each washing cycle was determined.

Preliminary clogging trials showed that undried clay (or fine sand) was easily washed away and that few particles were retained within the internal pores after a few washing cycles. Preliminary clogging trials also showed that the coarse sand was mainly retained on the sample surface. Considering that undried clay, fine sand, and coarse sand were hardly retained within the internal pores, only the samples clogged by difficult-to-wash dried clay and medium sand causing internal clogging were scanned with CT. Preliminary trials found that drying did not affect the clogging phenomenon of sand. Therefore, in medium sand-clogged samples, only dried sand-clogged samples were scanned with CT considering that drying removes the influence of water on CT imaging.

After drying but before the clogged permeability measurement, samples clogged by particles were first scanned with CT to obtain the vertical distribution of sediment (Fig. 5b). Moreover, to explore the influence of pressure washing on the vertical distribution of sediment, the samples were again scanned after all the washing cycles were performed (Fig. 5b). Before the second CT scan, a 50 °C oven was used to dry the samples for 24 h to eliminate the influence of water on CT imaging.

The suspension was produced by uniformly blending the particles and water in a glass, which was used to spread clay suspension on sample surface. Both the particles passing through the sample and a small number of particles remaining in this glass were collected after the suspension was spread. Additionally, the particles passing through samples were collected during the clogged permeability test and each washing cycle along with the subsequent permeability test, as shown in Fig. 5. All the collected particles were dried at 105 °C for 24 h and subsequently weighed to obtain the mass.

## Results and discussion

### Particle retention

The mass of the particles retained in sample pore structure was calculated according to the 240-g particle mass in the prepared suspension and mass of accumulated collected particles.

2 $${\text{M}}_{\text{n}}=240-{\text{C}}_{\text{a}}-{\text{C}}_{\text{c}}-\sum_{j=1}^{n}{\text{C}}_{\text{j}}$$

where *M*_*n*_ denotes the retained mass after completing the first *n* washing cycles along with the subsequent permeability tests; *C*_a_ denotes the collected mass from suspension addition to no particles flowing out of the sample bottom; *C*_c_ denotes the collected mass during the clogged permeability test; and *C*_*j*_ denotes the collected mass during washing cycle *j* as well as the subsequent permeability test.

**Figure 6:**
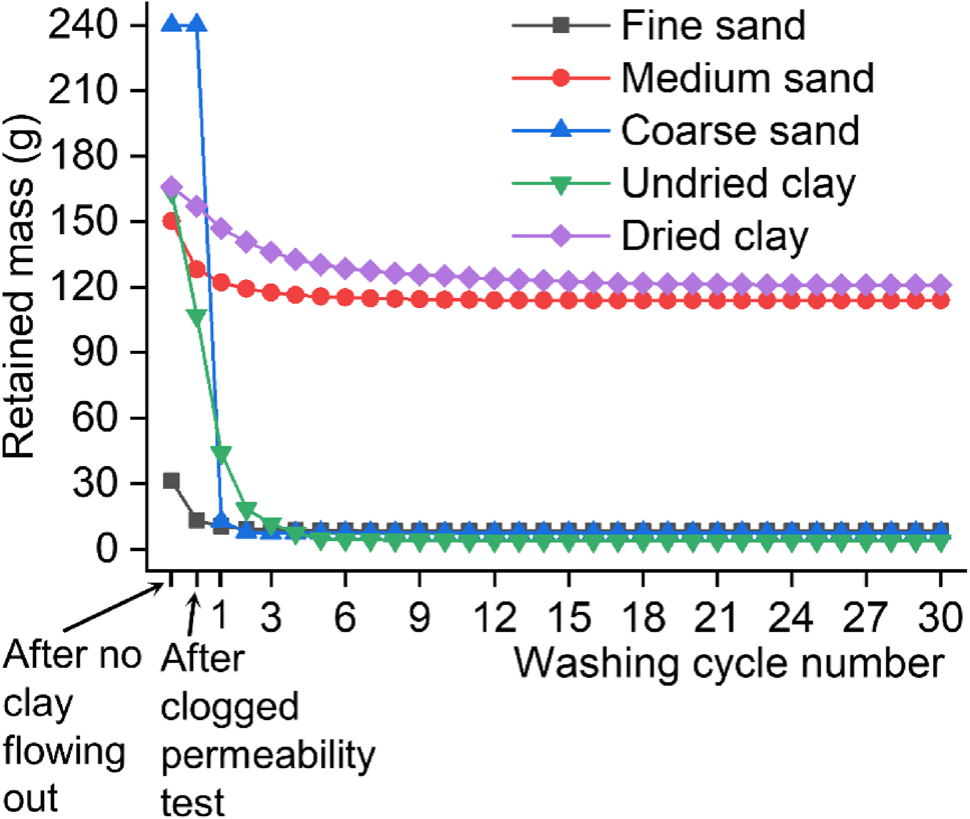
The mass of retained particles after completing different experimental steps.

Figure 6 shows the mass of retained particles after completing different experimental steps. Because the measurements indicate that drying does not affect the clogging phenomenon of sand, only the results of no drying-clogging experiments are shown in Fig. 6 for sand clogging. Figure 6 shows that washing cycles can partially remove the retained particles. But the removal effect of pressure washing is strongly related to particle size for sand clogging and drying degree for clay clogging (subsequent sections provide the details).

The mass of retained particles can also be calculated from the CT scan-determined volume and specific gravity. The relative difference (*D*) between the retained masses determined with CT scan (*m*_s_) and the collection method (*m*_c_) is defined as follows:

3 $${\text{D =}} \frac{{m}_{\text{s}}-{m}_{\text{c}}}{{m}_{\text{c}}}$$

The calculations show that all the relative differences range from −4.50 to 9.09%. These differences are not high, indicating that the retained mass determined with CT scan is generally consistent with that determined with the collection method and that high-resolution CT scan may be reliable in distinguishing the retained sand (or clay) from PC matrix.

### Permeability

**Figure 7:**
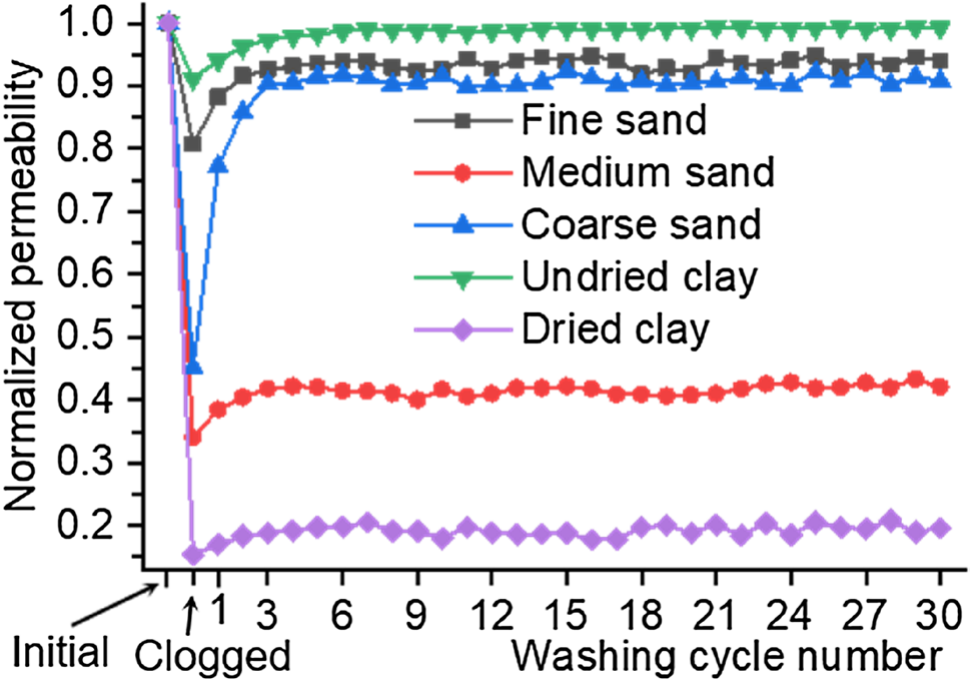
The normalized permeability versus the number of washing cycles.

**Figure 8:**
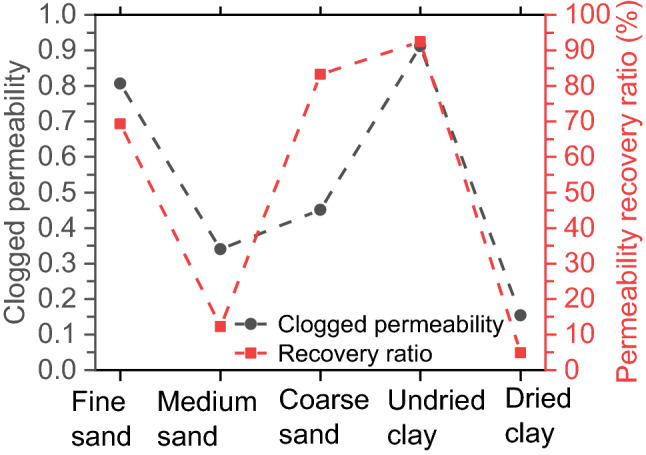
Clogged normalized permeability and permeability recovery ratio. The error bars represent the standard deviation.

Before and after the clogging as well as after each cycle of pressure washing, the permeability of each sample was measured. To place the measurements of all samples in a consistent framework, the permeability measurements of each sample are normalized to the initial unclogged value, as shown in Fig. 7. Figure 7 shows that the clogging reduces the permeability. Figure 7 also shows that the application of washing cycles can partially recover the permeability; hence, the ratio of permeability recovery (RA) is introduced and defined.

4 $$\text{RA} = \frac{{\text{k}}_{\mathrm{f}}-{\text{k}}_{\mathrm{c}}}{\text{1} - {\text{k}}_{\mathrm{c}}}$$

where ${\text{k}}_{\mathrm{c}}$ denotes the clogged permeability and ${\text{k}}_{\mathrm{f}}$ denotes the final permeability after completing all washing cycles. Figure 8 shows RA and the clogged normalized permeability, which are strongly related to particle size for sand clogging and drying degree for clay clogging (subsequent sections provide the details).

### Vertical sediment distribution

**Figure 9:**
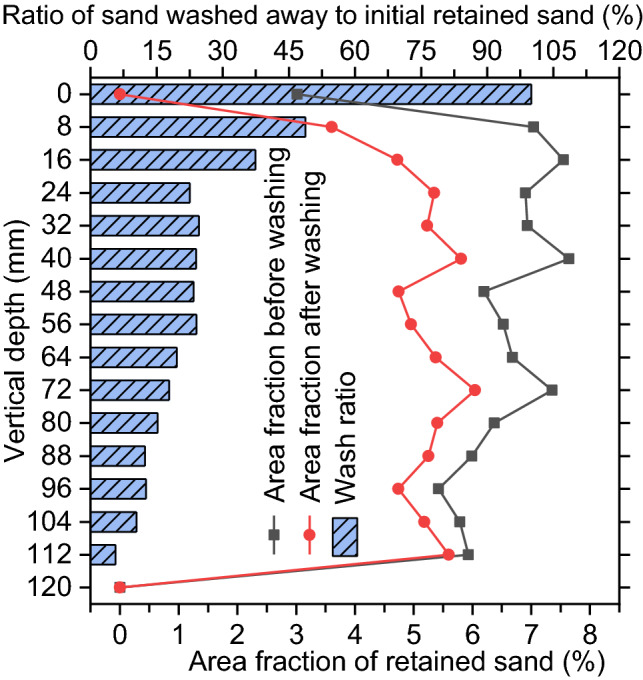
Vertical distributions for the area fraction of retained sand before any washing and after 30 washing cycles (line graph) and vertical distribution for the wash ratio of sand (bar graph) for the medium sand-clogged sample.

**Figure 10:**
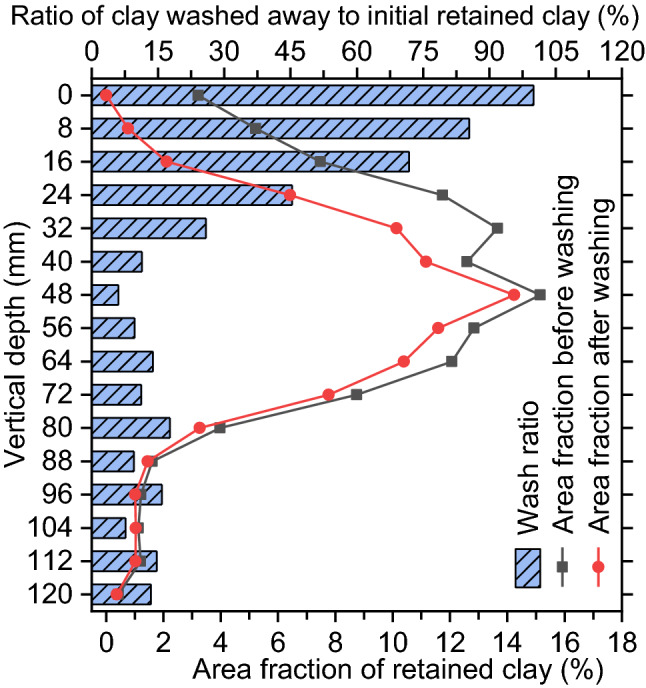
Vertical distributions for area fraction of retained clay before and after 30 washing cycles (line graph) and distribution of the wash ratio of clay (bar graph) for the dried clay-clogged sample.

Figure 9 shows the vertical distributions of sediment in the medium sand-clogged samples before any washing and after 30 washing cycles. In the slice images at the 16 vertical depths, the area of sand washed away is calculated by subtracting the final retained sand area (after 30 washing cycles) from initially retained sand area (before any washing). By dividing washed sand area by initially retained sand area, the ratio of sand washed away is determined. This ratio is referred to as the wash ratio of sand. The definition of the wash ratio of clay is similar. Figure 9 also shows the vertical distribution of the wash ratio of sand. In contrast, Fig. 10 shows the sediment distributions in dried clay-clogged samples before any washing and after 30 washing cycles and the distribution of the wash ratio of clay. By comparing Figs. 9 and 10, it is found that the retained clay mostly occurs within the upper region, while the sand particles are trapped throughout the entire sample before any washing. Clay particles have soil cohesion and easily adhere to the pore wall within the upper region. In contrast, sand particles do not have soil cohesion and can easily enter the lower region. “Sand-clogging and clay-clogging mechanisms” provides the detailed differences between sand-clogging and clay-clogging mechanisms.

### Sand clogging

For fine sand-clogged samples, the clogged normalized permeability is as high as 0.807, meaning that the clogged permeability is 80.7% of the unclogged permeability. Moreover, only three washing cycles almost recover the permeability to the initial value, with a post-washing normalized permeability of 0.927, as shown in Fig. 7. Similarly, the mass of retained particles is very low (approximately 8.96 g) after only three washing cycles (Fig. 6). These results indicate that fine sand is readily carried through PC. This mainly occurs because the particle size of fine sand is smaller than the sample pore size.

For coarse sand-clogged samples, no particles passing through the samples are found, whereas many particles accumulating on the sample surface are observed during the addition of coarse sand suspension and the clogged permeability test. After only three washing cycles remove almost all accumulation particles (pressurized water forces them off the surface), the permeability nearly recovers to the initial value, with a post-washing normalized permeability of 0.904 (Fig. 7). These findings indicate that coarse sand tends to be trapped on or in the sample surface because the particle size of coarse sand is larger than the sample pore size.

The retained mass quickly declines during the clogged permeability test and the first 3 washing cycles but subsequently declines very slowly for the medium sand-clogged samples, with a final retained mass as high as 113.88 g, as shown in Fig. 6. The pore network in PC is heterogeneous and complex, and large and small pores all exist^7^. Some particles retained in large pores are readily washed away. After the first 3 washing cycles wash away these sand particles, the remaining particles retained within the small pores (especially narrow pore necks) are very difficult to wash away. Therefore, few particles passing through the samples are collected during the last 27 washing cycles.

Figure 7 shows that the clogging of medium sand causes a sharp reduction in permeability, with a clogged normalized permeability of 0.340. Applying washing cycles partially recovers the permeability. However, only the first three washing cycles produce a small amount of recovery, and the subsequent washing cycles have little effect; as a result, the permeability recovery ratio of all 30 washing cycles is only 12.29%. These results indicate that medium sand causes serious, difficult-to-recover clogging.

The vertical distribution of sediment in the medium sand-clogged sample before any washing shows that the particles are trapped throughout the entire sample (Fig. 9), indicating that the medium sand causes full-depth clogging. However, note that a few particles are trapped near the top or bottom surface. This may be because boundary effects, causing the near-surface regions to have an increasing number of pores, make it difficult to retain the particles.

Figure 9 also shows the vertical distribution of sediment in the medium sand-clogged sample after 30 washing cycles and the vertical distribution for the ratio of sand washed away. The average wash ratio is only 24.91%, which is obtained by averaging the 16 wash ratios from the 16 images of one sample. Similarly, the 113.88-g retained mass still existed even after 30 washing cycles. These findings indicate that medium sand causes difficult-to-recover clogging.

As shown in Fig. 9, the wash ratio generally decreases with the increasing vertical depth because pressure washing has a higher scouring force within the near-surface region. Because of buffering effect in the near-surface region, the scouring force declines when washing water flows through the region far from sample surface. Additionally, some particles washed away from the upper region may be trapped within the lower region during pressure washing. Therefore, the lower region shows a lower wash ratio.

### Clay clogging

#### Undried clay

For the undried clay-clogged samples, the clogged normalized permeability is as high as 0.912, meaning that the clogged permeability is 91.2% of the unclogged permeability. Moreover, only five washing cycles almost recovered the permeability to the initial value, with a post-washing normalized permeability of 0.984 (Fig. 7). Similarly, as shown in Fig. 6, the retained mass of clay shows a very low value (approximately 4.87 g) after only 5 washing cycles. These results indicate that pressure washing readily washes away the undried clay retained within the sample pores. This is mainly due to low cohesion of undried clay and clay particles being much smaller than sample pores.

#### Dried clay

Figure 6 shows that 120.96-g retained mass for dried clay is considerably higher than 4.01-g retained mass for undried clay after all 30 pressure washing cycles, indicating that the retained dried clay is considerably harder to wash away than the retained undried clay. This is mainly due to drying increasing soil cohesion and bond formation^26,27^. Specifically, drying increases the cohesion of internal clay particles and clay adhesion to pore wall of PC (details are presented in “Clay-clogging mechanism”).

Figure 6 shows that the retained mass declines rapidly in the early stage (the first 5 washing cycles), while it then declines very slowly for dried clay-clogged samples. The pore network of PC is heterogeneous and complex, and the tortuosity in different pore channels differs^7^. Some particles, especially in low-tortuosity pore channels, are readily washed away because of the small adhesion force to pore wall of PC. After the first 5 washing cycles wash away these sand particles, water flow is hard to wash away remaining stably adhered particles, especially in high-tortuosity pore channels. The particles trapped within narrow pore necks are harder to wash away. Thus, a few particles carried through PC were collected during the last 25 washing cycles.

Figure 7 shows that the clogging of dried clay sharply reduces the permeability. Applying washing cycles partially recovers the permeability. However, the amount of recovery appears pretty small, especially during the last 25 washing cycles. The permeability recovery ratio of all 30 washing cycles is only 4.91%.

Figure 10 shows the vertical distribution sediment in PC clogged by dried clay before any washing. More than 77% of the total retained clay occurs within the vertical region 24–72 mm below the sample surface, while less than 23% occurs within the remaining region. This result is generally consistent with a previous study^25^. The clay suspension spread on the sample surface flowed into the interior at a high infiltration velocity and seepage force; thus, the near-surface region retained a few particles. This process is very consistent with sediments-carrying, high-velocity runoff entering PC pavement under field conditions. Because of buffering effect in the near-surface region, the infiltration velocity decreases when clay suspension flows through the region far from sample surface; hence, the particles are mostly trapped within the vertical region 24–72 mm below the surface. However, little clay is retained within the lowermost region 80–120 mm below the surface. This may be due to the filtering of the upper region^32^.

Figure 10 also shows the sediment distribution in dried clay-clogged PC after 30 washing cycles and the distribution of the wash ratio of clay. The wash ratios near the sample surface are considerably higher than those within the region 40–120 mm below the surface. Quantitatively, the average wash ratio within the region 0–32 mm below the surface is as high as 65.66%, whereas the ratio within the region 40–120 mm below the surface is only 11.90%. This mainly occurs because pressure washing with the high scouring force readily washes away retained particles near the surface. Because of buffering effect in the near-surface region, the scouring force declines when washing water flows through the region far from sample surface; hence, the wash ratio far from sample surface is lower.

The average wash ratio of 28.70% is obtained by averaging the 16 wash ratios from the 16 images of one sample. In contrast, the permeability recovery ratio (RA) is only 4.91%. The comparison indicates that RA remains very low, although some clay particles are washed away. This is because of the effective permeability in PC (a porous medium) depending mainly on the lowest-permeability layer^33^. The most clogging occurs at a depth of 48 mm from the surface among the 16 vertical depths (Fig. 10), indicating that the lowest-permeability layer occurs at a depth of approximately 48 mm after the clogging. As expected, the wash ratio of 6.01% at the 48-mm depth is lowest among the 16 vertical depths. Thus, the permeability recovery ratio shows a low value of 4.91%. These measurements indicate that washing water has difficulty washing away the clay that is retained within the lowest-permeability layer; thus, despite some particles being washed away, the samples exhibit low permeability recovery ratios.

### Sand-clogging and clay-clogging mechanisms

#### Sand-clogging mechanism

The measurements indicate that drying does not affect the clogging phenomenon of sand. Regardless of whether the retained sand undergoes drying, the fine sand of 0.075–0.25 mm is readily carried through the samples, and the coarse sand of 0.5–1 mm tends to be trapped on or in the sample surface for PC applied in this study. In contrast, the medium sand of 0.25–0.5 mm is trapped throughout the entire sample, causing serious, full-depth, difficult-to-recover clogging. Therefore, it can be concluded that the clogging patterns of sand depend mainly on sand particle sizes relative to PC pore sizes, as previously reported^7,18^. According to sand particle sizes relative to PC pore sizes, the clogging patterns of sand are classified into easy-passing clogging, surface clogging, and full-depth clogging, corresponding to sand clogging of 0.075–0.25, 0.5–1, and 0.25–0.5 mm in this study, respectively.

**Figure 11:**
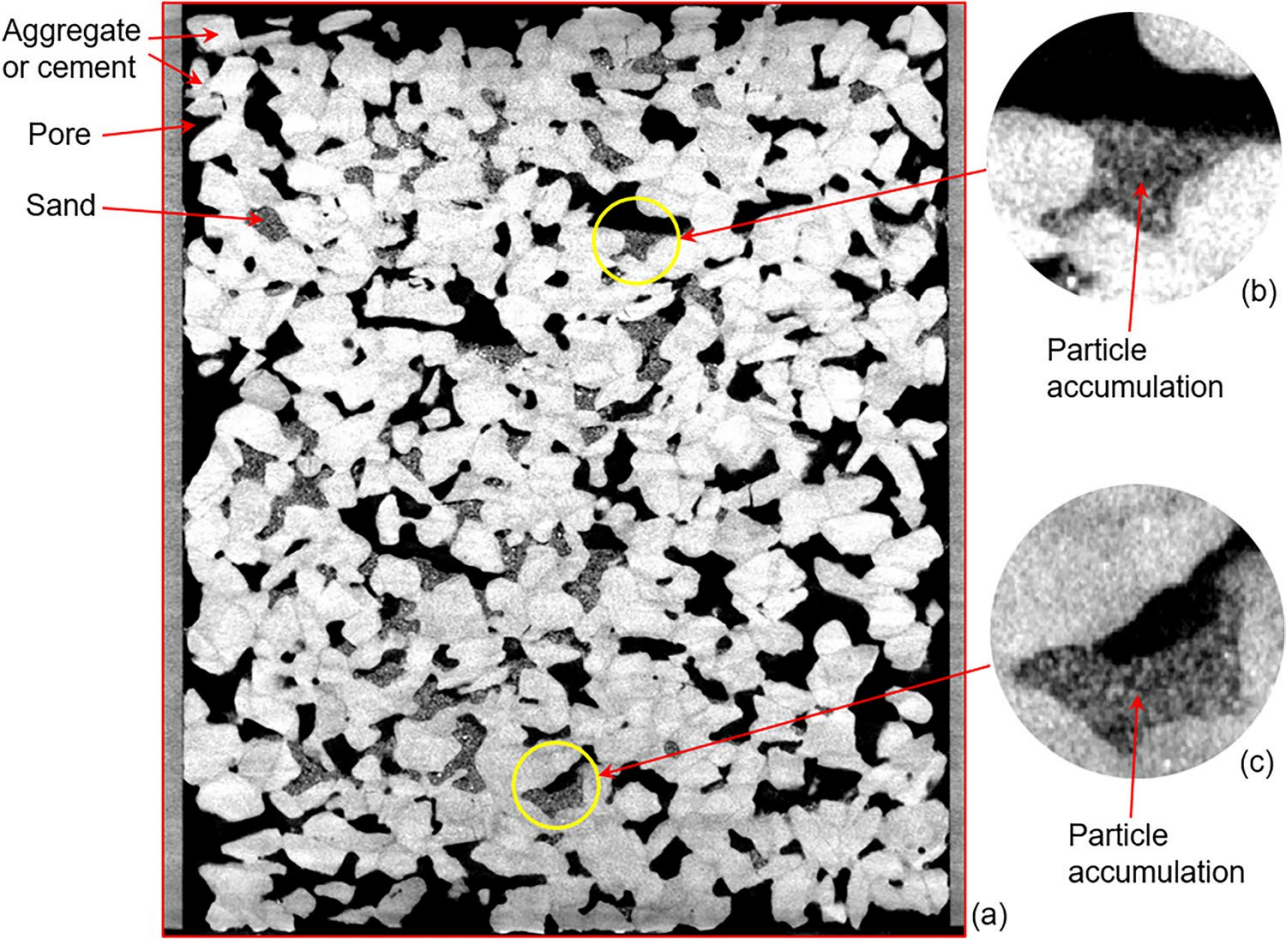
The CT image of the medium sand-clogged sample after all 30 washing cycles: (**a**) vertical image; (**b**) particle accumulation-clogging; and (**c**) particle accumulation-clogging.

Figure 11a shows the vertical CT image of a medium sand-clogged sample after 30 washing cycles. When water-carrying particles flow through a narrow pore neck whose size is smaller than the size of one of the particles, this particle will be trapped on this neck; next, more particles accumulate at this location until the pore is completely filled, thus forming particle accumulation clogging, as shown in Fig. 11b,c.

#### Clay-clogging mechanism

In contrast to sand clogging, the clogging of clay, whose particles are much smaller than PC pores, depends more on its physical properties, particularly its cohesiveness^19,25^. However, the measurements indicate that pressurized water is easy to wash away the undried clay retained within the sample pores. This may occur because the scouring force of water flow during pressure washing cycles exceeds the cohesion of internal clay particles and clay adhesion to pore wall of PC.

**Figure 12:**
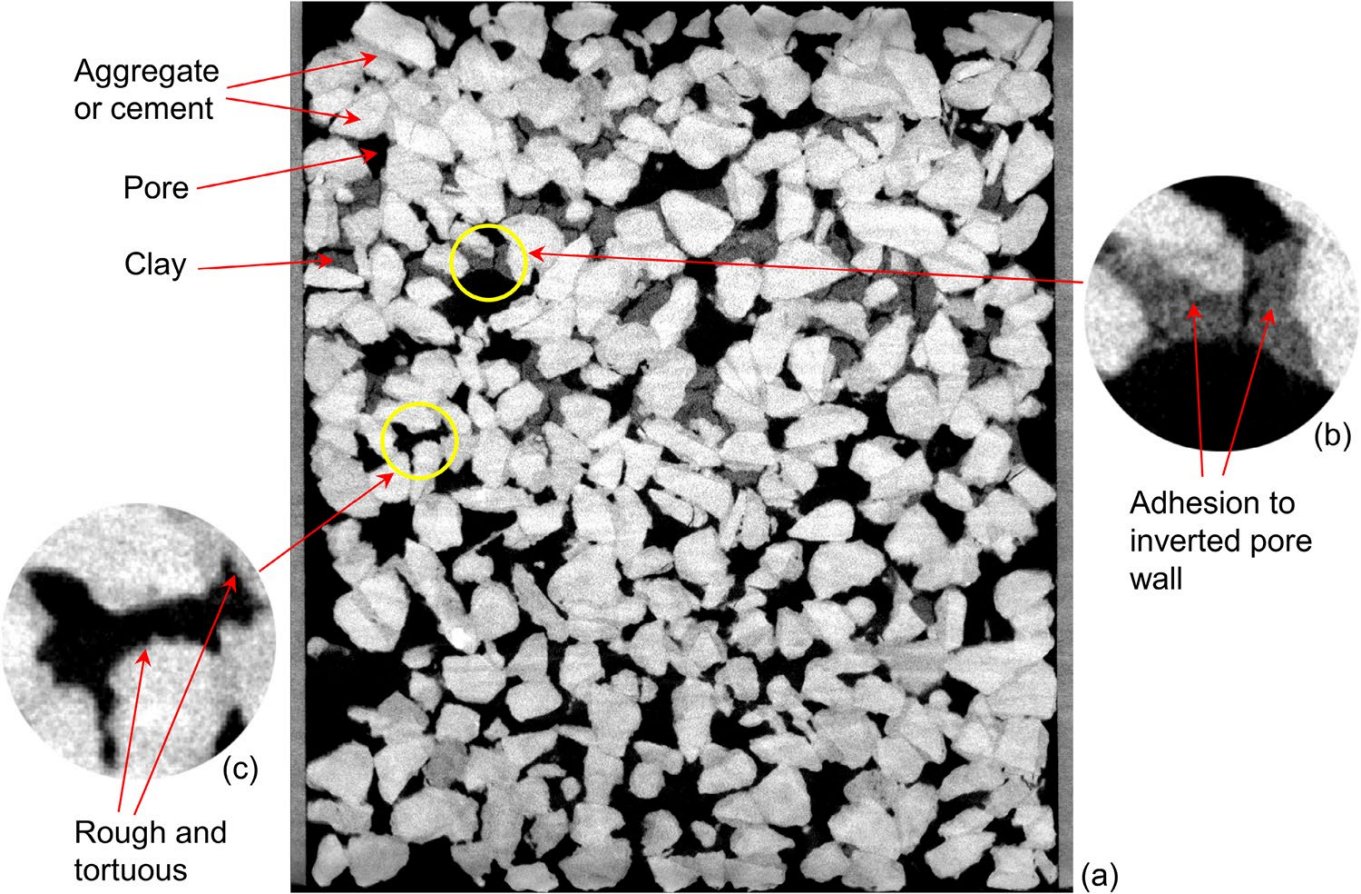
The CT image of the dried clay-clogged sample after all 30 washing cycles: (**a**) vertical image; (**b**) particles adhering to inverted pore wall; and (**c**) rough tortuous pore wall.

However, numerous studies have found that drying increases soil cohesion and bond formation^26,27^. Thus, the cohesion of internal clay particles and clay adhesion to pore wall of PC probably increase after drying, as reported by a previous study^25^. Fan, et al.^34^ have reported that clay may resist erosion when the flow velocity is below 100 cm/s. In contrast, the infiltration velocities are lower than 2 cm/s, even in unlogged samples. Infiltration with a low velocity typically produces a low scouring force; hence, the scouring force may appear lower than the cohesion of internal clay particles and clay adhesion to pore wall of PC. As a result, the retained particles are difficult to wash away, although adhering to the inverted pore wall, as shown in Fig. 12a–c.

In addition to drying increasing soil cohesion and bond formation, the rough, tortuous property of PC pore wall plays an important role in the hard-to-recover clogging caused by dried clay. The pore wall shows a very rough, tortuous property because PC randomly consists of cementing materials and aggregates with varying shapes, as seen in Fig. 12d. This property may enhance clay adhesion to the pore wall and make it more difficult to remove dried clay adhering to the pore wall.

### Novelty of this study and Implications for pavement maintenance

Previous studies have also focused on the clogging of PC. However, most previous studies investigated only particle size-related clogging, especially sand clogging^12–15^. A few previous studies focused on only the clay clogging^19,25^. To our best knowledge, to date, no study has made in-depth comparisons between sand and clay clogging mechanisms of PC; no study has made quantitative comparisons between vertical sediment distributions of sand-clogged and clay-clogged PCs. This study comparatively investigated sand and clay clogging mechanisms of PC and vertical sediment distributions of sand-clogged and clay-clogged PCs. Moreover, the results can provide evidence and implications for pavement maintenance.

As shown in Fig. 8, the clogged normalized permeability of 0.154 and permeability recovery ratio of 4.91% in dried clay-clogged samples are lowest among all samples. This very low recovery ratio still occurs even after 30 washing cycles as well as the subsequent permeability tests. These results indicate that dried clay causes the most serious clogging and that the permeability is most difficult to recover. Therefore, in field conditions, clay-carrying runoff water in combination with hot, sunny conditions should receive special attention.

However, the clogging effect of dried clay on the permeability of PC can be readily eliminated by using pressure washing before hot, sunny conditions dry the retained clay. The measurements in undried clay-clogging experiments clearly indicate that undried clay retained in sample pores is easily washed away. Therefore, the retention and accumulation of clay particles will not occur if pressure washing is used to clean clogged PC pavement before retained clay dries. From this perspective, in practice, it is recommended to apply pressure washing to clean PC pavement before it is subjected to hot sunny conditions. Using this method, the effect of drying-related clay clogging can be eliminated.

For PC applied in this study, the fine sand of 0.075–0.25 mm, coarse sand of 0.5–1 mm, and medium sand of 0.25–0.5 mm cause easy-passing clogging, surface clogging, and full-depth clogging, respectively. The clogged normalized permeability of 0.354 and permeability recovery ratio of 12.90% in the medium sand-clogged samples are much lower than those of the other two types of sand, as shown in Fig. 8. Therefore, in practice, regarding the particle-related sand clogging, the sediment particles that will cause full-depth clogging should receive much more attention than the particles causing the other two clogging patterns.

## Conclusions

This study comparatively investigated sand and clay clogging mechanisms of PC and vertical sediment distributions of sand-clogged and clay-clogged PCs. Clay and three sizes of sand were used to clog PC under two exposure methods (no drying and drying). CT was used to scan the clogged samples before and after 30 pressure washing cycles. The clogged permeability and permeability after each washing cycle were measured. This study provides evidence for developing effective pavement maintenance strategies. The following conclusions can be drawn:

1. The clogging patterns of sand depend mainly on sand particle sizes relative to PC pore sizes. Applied fine sand of 0.075–0.25 mm is readily carried through the samples, and coarse sand of 0.5–1 mm tends to be trapped on or in the sample surface for the applied PC. In contrast, medium sand of 0.25–0.5 mm is trapped throughout the entire sample. Thus, the patterns of particle-related sand clogging are classified into easy-passing clogging, surface clogging, and full-depth clogging.
2. The clogged normalized permeability of 0.354 and permeability recovery ratio of 12.90% in the medium sand-clogged samples are much lower than those for the other two types of sand. Therefore, regarding particle-related clogging, medium sand that will cause serious, full-depth, difficult-to-recover clogging should receive much more attention than fine and coarse sand.
3. Pressure washing readily washes away the retained undried clay owing to low cohesion of undried clay and small size of clay particles. Only five washing cycles almost recover the permeability to the initial value, with a post-washing normalized permeability of 0.984. Similarly, the mass of retained particles approaches 0 after only five washing cycles.
4. More than 77% of the total retained clay occurs within the vertical region 24–72 mm below the sample surface after clay clogging. Moreover, the most clogging (the lowest-permeability layer) occurs at a depth of approximately 48 mm after clay clogging. For samples clogged by dried clay, the permeability recovery ratio is very low because the clay retained within the region 40–120 mm below the surface (especially within the lowest-permeability layer) is hard to wash away. The average wash ratio is 28.70%, whereas the permeability recovery ratio and the minimum wash ratio are only 4.91% and 6.01%, respectively.
5. The clogged normalized permeability of 0.154 and permeability recovery ratio of 4.91% in dried clay-clogged samples are lowest among all the samples, indicating that dried clay causes the most serious, hardest-to-recover clogging, which is mainly because of drying increasing the cohesion of internal clay particles and clay adhesion to pore wall of PC. The adhesion-clogging phenomenon is strengthened by the rough, tortuous property of the pore wall. However, the clogging effect of dried clay on the long-term performance of PC can be readily eliminated by using pressure washing before the retained clay is dried.

In practice, regarding the particle-related sand clogging, the sediment particles that will cause full-depth clogging should receive much more attention than the particles causing easy-passing clogging and surface clogging patterns. Regarding the drying-related clay clogging, it is recommended that pressure washing is used to eliminate the clogging effect of dried clay before hot, sunny exposure conditions dry the retained clay.

Future studies are required to measure the vertical sediment distributions of a wider range of samples, especially exposed to real field conditions. In addition to sand clogging and clay clogging, PC clogging by organic matter from surrounding vegetation should receive attention in future studies.
